# Body‐ and Movement‐Oriented Interventions for Posttraumatic Stress Disorder: A Systematic Review and Meta‐Analysis

**DOI:** 10.1002/jts.22465

**Published:** 2019-10-28

**Authors:** Minke M. van de Kamp, Mia Scheffers, Janneke Hatzmann, Claudia Emck, Pim Cuijpers, Peter J. Beek

**Affiliations:** ^1^ Specialized Centre for Trauma Treatment of PsyQ The Hague The Netherlands; ^2^ School of Human Movement and Education Windesheim University of Applied Sciences Zwolle The Netherlands; ^3^ Faculty of Behavioural and Movement Sciences Vrije Universiteit Amsterdam Amsterdam The Netherlands

## Abstract

To assess the efficacy of body‐ and movement‐oriented interventions (BMOIs) in traumatized adults with posttraumatic stress disorder (PTSD), we conducted a systematic review and meta‐analysis of pertinent literature. Four bibliographical databases (PsycINFO, Ovid MEDLINE(R), EMBASE, and the Cochrane Central Register of Controlled Trials) were searched using keywords and text words for trials on BMOIs addressing PTSD. The search included articles published between October 2005 and August 2017. Studies were included if participants were adults suffering from PTSD, if BMOIs were the therapeutic strategy under investigation, and if a psychometrically evaluated standardized outcome measure for PTSD was used. No limitations for control conditions were applied. Hedges’ *g* was computed as the effect size (ES) for the treatment versus control condition. The meta‐analysis included 15 studies, which resulted in a mean ES of *g* = 0.85, 95% CI [0.31, 1.39], with very high heterogeneity, *I* ^2^ = 91%. After removing one study as outlier, a mean effect size of *g* = 0.56, 95% CI [0.29, 0.82] (i.e., medium effect), still with considerable heterogeneity, *I* ^2^ = 57%, was found. BMOIs seem to be effective in reducing symptoms of PTSD, but more research is needed to identify working mechanisms and to determine which types of intervention are most effective for various subgroups of patients.

Posttraumatic stress disorder (PTSD) is a chronic and debilitating disorder characterized by symptoms of reexperiencing, avoidance, emotional numbing, and hyperarousal as a consequence of one or more traumatizing experiences (American Psychiatric Association [APA], 2013). The disorder is associated with suicidal ideation and behavior (Ying et al., 2015; Youssef et al., 2013), as well as high rates of comorbid psychiatric disorders, including mood and anxiety disorders, somatoform disorders, and substance use disorders (Jacobi et al., 2004; Yehuda et al., 2015). Additionally, PTSD is associated with medical comorbidities (such as cardiovascular, respiratory, musculoskeletal, neurological, and gastrointestinal disorders), chronic pain and inflammation, diabetes, and metabolic syndrome (Gupta, 2013; Yehuda et al., 2015).

Various forms of treatment, such as exposure, Eye Movement Desensitization and Reprocessing (EMDR), and cognitive behavioral interventions, have been shown to be effective in the treatment of PTSD (Bisson, Roberts, Andrew, Cooper, & Lewis, 2013; Cloitre et al., 2011). However, treatment dropout rates are high, and posttreatment, substantial residual PTSD symptoms often remain (Bradley, Greene, Russ, Dutra, & Westen, 2005; Corrigan & Hull, 2015), including sleep disturbances (Pruiksma et al., 2016; Zayfert & DeViva, 2004), pain and psychosomatic pain, and other physical health problems (Galovski, Monson, Bruce, & Resick, 2009; Shipherd, Clum, Suvak, & Resick, 2014).

PTSD involves a fundamental dysregulation of arousal modulation (Van der Kolk, 2015) and is associated with significant problems in body‐ and self‐awareness as well as affect regulation (Lanius, Bluhm, & Frewen, 2011; Pole, 2007; Schauer & Elbert, 2010). Neurobiological research suggests that the evolutionarily older brain systems that play a central role in processing overwhelming stress are not reached sufficiently through verbal and cognitive interventions (Ogden, Pain, & Fisher, 2006; Van der Kolk, 2015). These interventions primarily address the prefrontal cortex, and this evolutionarily newest part of the brain is not able to influence the “lower brain areas” in traumatized people (Van der Kolk, 2015). Therefore, for patients with PTSD, a bottom‐up approach, starting with the body and physical sensations, may be a more appropriate form of treatment in facilitating the regulation of arousal and affect (Levine, 2010; Ogden et al., 2006; Van der Kolk et al., 2014).

Body‐ and movement‐oriented interventions (BMOIs), such as sensorimotor psychotherapy (Langmuir, Kirsh, & Classen, 2012; Ogden et al., 2006), somatic experiencing (Levine, 2010), body‐oriented psychotherapy (BOP; Röhricht, 2009), and psychomotor therapy (Probst, Knapen, Poot, & Vancampfort, 2010), provide such a bottom‐up approach and may form a valuable addition to current cognitive behavioral and exposure‐based treatments. Body‐ and movement‐oriented interventions can be defined as those in which physical activity and corporeality are the central themes and core focus of the intervention; they are characterized by their use of movement activities and focus on bodily experiences (Probst et al., 2010). Methods derived from sports as well as from more body‐oriented approaches, such as relaxation therapy and body awareness therapy, are subsumed under this definition. Röhricht (2009) created an overview of the field of therapies that explicitly employ body‐oriented, nonverbal techniques. The BMOIs aim at decreasing PTSD symptomatology and increasing psychosocial and physiological well‐being by enhancing body awareness and integrating cognitive, affective, and somatic processing (Probst et al., 2010; Röhricht, 2009). These aspects are considered important in achieving effective emotion regulation and processing of traumatic experiences (Langmuir et al., 2012; Ogden et al., 2006).

In the last few decades, BMOIs for PTSD have received more attention in the literature as well as in clinical practice (Chan, Chan, & Ng, 2006; Emerson, Sharma, Chaudhry, & Turner, 2009; Ogden et al., 2006; Stankovic, 2011). These interventions are described as clinically relevant, feasible, and acceptable for patients suffering from PTSD (Clark et al., 2014; Grodin, Piwowarczyk, Fulker, Bazazi, & Saper, 2008; Price, McBride, Hyerle, & Kivlahan, 2007; Stankovic, 2011).

With the growing attention on BMOIs for PTSD, more studies have been published on the effectiveness of these approaches, which brings along a need for a systematic review and meta‐analysis of these studies. The purpose of this article was, therefore, to review the results of research on BMOIs in traumatized adults and to draw conclusions about their effectiveness in decreasing PTSD symptoms (primary outcome) as well as comorbid symptoms of depression (secondary outcome). Only studies in which BMOIs were administered on their own, without being combined with other types of intervention, were included. As BMOIs are relatively novel in the treatment of PTSD, we included a broad range of studies that match the definition given earlier to assess the effects of these interventions. Based on the results of the search, subcategories of interventions were formed where feasible.

## Method

### Eligibility Criteria and Search Procedure

For the present systematic review, included articles had to (a) have been published in English, German, Dutch, or French; (b) have had their abstract published in English; (c) have been published in a peer‐reviewed journal; (d) focus on traumatized adults, aged at least 18 years, with a primary diagnosis of PTSD; (e) include BMOI therapy (as defined and described by Probst et al., 2010 and Röhricht, 2009) as one of the investigated therapeutic strategies; (f) be designed to include a comparison outcome trial with a control group or single group pre–post comparison trial; and (g) involve a standardized outcome measure for PTSD, which was evaluated psychometrically. There were no restrictions as to the make‐up of the control groups. For inclusion in the meta‐analysis, studies also had to be randomized controlled trials (RCTs) or non‐randomized controlled studies and had to provide enough data to calculate effect sizes. Searches were conducted in the following databases: PsycINFO, Ovid MEDLINE(R), EMBASE, and the Cochrane Central Register of Controlled Trials. From selected studies, cross‐references were checked. The last search was performed on November 6, 2017. Search terms included body‐ and movement‐oriented modalities and terms related to PTSD. Search results were limited to studies of adult samples, the languages mentioned in the eligibility criteria, and journal articles. A full electronic search strategy is presented in the Supplementary Materials.

### Study Selection

The first, second, and third authors independently screened and selected journal articles identified by the search strategy for inclusion in the study based on the aforementioned inclusion and exclusion criteria. First, titles and abstracts were screened for eligibility. Second, each author independently examined the full text of all studies he or she considered to be eligible and created a list of proposed studies to be included. Any disagreement was resolved by discussion until consensus was reached between the three authors.

### Data Collection Process and Data Analysis

The first and third authors independently extracted data using a standardized format that contained information on participants (age, gender, severity of PTSD, military or civilian), interventions and comparisons (description of intervention, description of comparison, dose and length of treatment), outcomes (measures used, timing of administration, psychometric properties, results), and study design (single group or between groups, follow‐up, method of allocation and randomization). When any of these elements were missing or unclear, the study's authors were contacted.

#### Risk of bias in individual studies

Risk of bias in individual studies was assessed with the Cochrane Risk of Bias Tool (Higgins, 2008). The Cochrane Risk of Bias Tool provides criteria to assess the risk of bias in individual studies based on six domains: sequence generation (randomization process); allocation concealment; blinding of participants, personnel, and outcome assessors; incomplete outcome data; selective outcome reporting; and other potential threats to validity. For the domain “blinding of participants, personnel and outcome assessors,” it was taken into account that a double‐blind methodology is virtually impossible in studies with psychological treatment as it is clear to participants what treatment they are receiving and to therapists which therapy they are offering. However, a well‐designed study should have ensured blinding of the assessor of outcome measures or have used outcome measures that are less likely to be influenced by lack of blinding in participants (e.g., physiological outcomes or self‐report).

#### Summary of measures and synthesis of results

A qualitative synthesis of all included studies was made in an overview containing study characteristics, such as description of participants, interventions, controls, outcome measures, and study design. For the quantitative synthesis, we used Comprehensive Meta‐Analysis (CMA; Version 3.0) to calculate pooled mean effect sizes. Effect sizes were computed as Hedges’ *g* using the random‐effects model for PTSD symptom severity and for measures of (comorbid) depression. Hedges’ *g* was selected as it corrects for sample size bias, which was deemed important as we expected some of the studies in this meta‐analysis to be based on small samples. We used the mean difference score between pretreatment and posttreatment for the treatment versus control condition. This method was preferred to standardization using posttreatment scores because both nonrandomized and randomized trials, which could contain pretest intervention differences, were included. For this calculation, a correlation between pretest and posttest data is needed. For studies that did not report this correlation, we imputed a conservative value of *r* = .7, as recommended by Rosenthal (1991). For the primary and secondary outcome measures, for cases in which both total scale and subscale scores for a measure were reported, we used the total scale score. If only subscale data were reported, we aggregated these subscale data to a total score according to the assessment manuals. If both self‐report and clinical interview were reported as an outcome measure, we combined the data from both measures in CMA. Furthermore, we included the studies in which follow‐up data were collected and reported for at least 1 month after posttest in a meta‐analysis for follow up, using the same effect size calculation as the previous analysis. Heterogeneity of effect sizes was assessed using the Q test and *I* ^2^. The Q test examines whether observed effect sizes vary significantly more than what would be expected on the basis of chance alone. A significant result of the Q test is evidence for heterogeneity. *I* ^2^ is a measure of inconsistency in meta‐analysis, which is considered an indicator of the heterogeneity in percentages.

#### Publication bias

For the examination of publication bias, asymmetry in the funnel plot was tested using Egger's test of the intercept (Egger, Smith, Schneider, & Minder, 1997). If the result of this test is significant, there is an asymmetry in the funnel plot, which is indication of publication bias. Furthermore, Duval and Tweedie's trim‐and‐fill method (Duval & Tweedie, 2000) was used to estimate how many studies were missing. If there are missing studies, Duval and Tweedie's trim‐and‐fill method imputes these studies, and, after imputation, estimates a new effect size.

#### Subgroup analyses

Four subgroup analyses were performed, using the mixed effects model. In the first subgroup analysis, the studies with a low risk of bias were compared with the studies with a high risk of bias. For this analysis, we considered studies with no more than one unclear area on the Cochrane Risk of Bias tool as studies with a low risk of bias. In the second subgroup analysis, studies with veterans as participants were compared to studies with civilians as participants. In the third subgroup analysis, waitlist control conditions and active control conditions (i.e., psychoeducation or “treatment as usual”) were compared. Because the variation in the interventions was large, a fourth subgroup analysis was conducted, involving a comparison between yoga and the collective of all other BMOIs included in the study (see also Cramer, Anheyer, Saha, & Dobos, 2018).

In the main analysis, the effect size based on the mean difference score was calculated. Therefore, a correlation between pretest and posttest data was needed, and, as stated before, we imputed a value of *r* = .7 for studies that did not report such correlation. We performed sensitivity analyses with *r* values of .5 and .3. Furthermore, a sensitivity analysis was performed calculating the effect size based on the posttreatment group difference. Finally, a separate mean effect size for the RCT included in the meta‐analysis was calculated. All methods were executed according to the PRISMA guidelines (Moher, Liberati, Tetzlaff, Altman, & Group, 2009) for the conduct and reporting of systematic reviews.

## Results

### Study Selection

**Figure 1:**
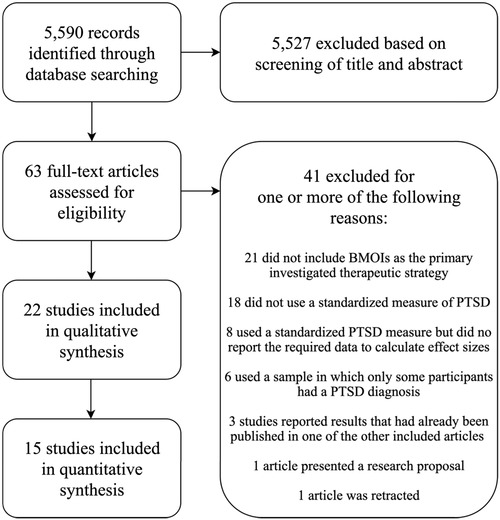
Flowchart of the inclusion of studies. PTSD = posttraumatic stress disorder; BMOI = body‐ or movement‐oriented intervention.

Figure 1 displays the flow of information through the phases of the systematic review. We identified 5,590 records, after removing the duplicates, through database searching. In the initial screening of abstracts, 5,527 records were excluded. We assessed 63 full‐text articles for eligibility, and 41 articles were excluded because they did not meet all eligibility criteria for one or more of the reasons displayed in Figure 1. Finally, 22 articles were included in the qualitative synthesis, 15 of which were included in the meta‐analysis.

### Study Characteristics

The selected studies included both civilian participants and veterans as well as both men and women. A total of 11 studies involved a form of yoga (Carter et al., 2013; Descilo et al., 2009; Jindani, Turner, & Khalsa, 2015; McCarthy et al., 2017; Mitchell et al., 2014; Price et al., 2017; Seppälä et al., 2014; Staples, Hamilton, & Uddo, 2013; Thordardottir, Gudmundsdottir, Zoega, Valdimarsdottir, & Gudmundsdottir, 2014; Van der Kolk et al., 2014; Walker & Pacik, 2017), whereas the other studies involved a variety of other BMOIs. The control conditions were waitlist (Carter et al., 2013; Descilo et al., 2009; Jindani et al., 2005; Kahn, Collinge, & Soltysik, 2016; Kaiser, Gillette, & Spinazzola, 2010; Kim et al., 2013; Mitchell et al., 2014; Seppälä et al., 2014; Thordardottir et al., 2014), a form of psychoeducation (Nakamura, Lipschitz, Landward, Kuhn, & West, 2011; Nakamura et al., 2017; Van der Kolk et al., 2014), and treatment as usual (TAU; Hoekenga, Thewissen, Bos, & Willemse‐van Son, 2010; Rosenbaum, Sherrington, & Tiedemann, 2014). One study compared a massage condition to massage and body‐oriented therapy (Price, 2005), and seven studies were single‐group studies without a control condition (Collinge, Kahn, & Soltysik, 2012; Gordon, Staples, He, & Atti, 2016; Manger & Motta, 2005; McCarthy et al., 2017; Price et al., 2017; Staples et al., 2013; Walker & Pacik, 2017). Outcome measures were self‐report measures for PTSD symptomatology as well as PTSD measures collected during clinical interviews; one study used a clinician‐administered measure of complex traumatic stress (Kaiser et al., 2010). The designs were RCTs (*n* = 12; Carter et al., 2013; Jindani et al., 2015; Kahn et al., 2016; Kaiser et al., 2010; Kim et al., 2013; Mitchell et al., 2014; Nakamura et al., 2011, 2017; Price, 2005; Rosenbaum et al., 2014; Van der Kolk et al., 2014), nonrandomized controlled studies (*n* = 3; Descilo et al., 2009; Hoekenga et al., 2009; Thordardottir et al., 2014), and single‐group studies (as already mentioned earlier). Detailed study characteristics are presented in Supplementary Table S1.

### Risk of Bias Within Studies

The risks of bias in each study as assessed with the Cochrane risk of bias tool are presented in Table 1. In 11 of the 22 studies (Carter et al., 2013; Jindani et al., 2015; Kahn et al., 2016; Kaiser et al., 2010; Kim et al., 2013; Mitchell et al., 2014; Nakamura et al., 2011, 2017; Price, 2005; Rosenbaum et al., 2014; Seppälä et al., 2014; Van der Kolk et al., 2014), an appropriate sequence generation was used. The allocation concealment was adequate in six studies (Carter et al., 2013; Kahn et al., 2016; Nakamura et al., 2011, 2017; Price, 2005; Rosenbaum et al., 2014). As mentioned in the Method section, a double‐blind design in which both the experimenter (therapist) and the participant (patient) are ignorant about the treatment is virtually impossible. One should take care, however, that either the individuals who assess the outcome are not informed about the allocation of the participants and/or that the outcome measures are less likely to be influenced by such information (physiological measures and self‐reports). All controlled studies except one (Descilo et al., 2009) met the latter criterion. Problems with regard to incomplete outcome data were solved satisfactorily in all controlled studies and most of the single‐group studies, except for the study by Price et al. (2017), which had a small sample size and an unexplained high dropout percentage, and the study by Manger and Motta (2005), in which the missing outcome data were directly related to the nature of the intervention and may have influenced the study results. In only 12 studies, follow‐up data after at least 1‐month posttest were gathered. In eight of these studies, the incomplete data were dealt with appropriately (Carter et al., 2013; Descilo et al., 2009; Kahn et al., 2016; Kaiser et al., 2010; Mitchell et al., 2014; Nakamura et al., 2017; Price, 2005; Seppälä et al., 2014). In only two studies (Rosenbaum et al., 2014; Van der Kolk et al., 2014), sufficient information was available to conclude that they were free of selective outcome reporting, whereas for the other studies, no pretest‐specified study protocol was available. The following other potential threats of validity were encountered: retrospective selection of participants in the study by Walker and Pacik (2017), a large baseline imbalance in the study by Kaiser et al. (2010), and a very short intervention and assessment period in the study by Nakamura et al. (2011).

**Table 1:** Risk of Bias for Included Studies

Study	Adequate Sequence Generation	Adequate Allocation Concealment	Blinding	Incomplete Outcome Data Addressed (short term)	Incomplete Outcome Data Addressed (long term)	Selective Outcome Reporting	Free of Other Bias
McCarthy et al. (2017)	−	−	−	+	?	?	+
Nakamura et al. (2017)	+	+	+	+	+	?	+
Price et al. (2017)	−	−	−	−	−	?	+
Walker & Pacik (2017)	−	−	−	+	?	?	−
Gordon et al. (2016)	−	−	−	+	−	?	+
Kahn et al. (2016)	+	+	+	+	+	?	+
Jindani et al. (2015)	+	?	+	+	?	?	+
Mitchell et al. (2014)	+	−	+	+	+	?	+
Rosenbaum et al. (2014)	+	+	+	+	?	+	+
Seppälä et al. (2014)	+	−	+	+	+	?	+
Thordardottir et al. (2014)	−	−	+	+	?	?	+
Van der Kolk et al. (2014)	+^a^	?	+	+	?	+	+
Carter et al. (2013)	+	+	+	+	+	?	+
Kim et al. (2013)	+	−	+	+	−	?	+
Staples et al. (2013)	−	−	+	+	?	?	+
Collinge et al. (2012)	−	−	+	+	?	?	+
Nakamura et al. (2011)	+	+	+	+	?	?	−
Hoekenga et al. (2010)	−	−	+	+	?	?	+
Kaiser et al. (2010)	+	−	+	+	+	?	−
Descilo et al. (2009)	−	−	?	+	+	?	+
Manger & Motta (2005)	−	−	−	−	−	?	+
Price (2005)	+	+	+	+	+	?	+

*Note*. + = Low risk of bias; ? = unclear; ‐ = high risk of bias.

a Based on correspondence with author.

### Results of Individual Studies and Synthesis of Results

**Figure 2:**
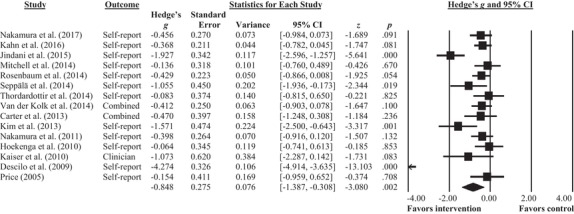
Meta‐analysis of all included studies.

The seven single‐group studies were not included in the meta‐analysis; six of these studies resulted in a significant decrease in overall PTSD symptomatology (Collinge et al., 2012; Gordon et al., 2016; McCarthy et al., 2017; Price et al., 2017; Staples et al., 2013; Walker & Pacik, 2017), and the seventh study only showed a significant decrease in hyperarousal symptoms (Staples et al., 2013). For the 15 studies included in the meta‐analysis, a random effects model effect size was calculated for all analyses because of the large heterogeneity in the interventions across the included studies (see Table S1). The random effects model meta‐analysis resulted in a mean effect size of *g* = 0.85, 95% CI [0.31, 1.39], which is considered a large effect (Lipsey & Wilson, 1993; see Figure 2). The test for heterogeneity resulted in *Q* = 155.9, *p* < .001, *I* ^2^ = 91%, indicating very high heterogeneity. The study by Descilo et al. (2009) was identified as an outlier as the range of the effect in the individual study was completely outside the range of the effect in the pooled studies. Therefore, we performed an additive meta‐analysis for all studies except that study. This resulted in a mean effect size of *g* = 0.56, 95% CI [0.29, 0.82], which is considered large (Lipsey & Wilson, 1993), with heterogeneity tests resulting in *Q* = 30.6, *p* = .004, *I* ^2^ = 57%.

For all studies that reported a follow‐up assessment performed at least 1 month posttest (*n* = 6; Carter et al., 2013; Kahn et al., 2016; Mitchell et al., 2014; Nakamura et al., 2017; Price, 2005; Seppälä et al., 2014), the mean effect size was *g* = 0.39, 95% CI [0.14, 0.65]. The test for heterogeneity resulted in *Q* = 2.02, *p* = .847, *I* ^2^ = 0%. The study by Kim et al. (2013) did not report follow‐up outcome data for the control group, and in the studies by Descilo et al. (2009) and Kaiser et al. (2010), the control groups received the investigated treatment during the follow‐up period and could therefore not be included in the follow‐up analysis. For the studies that reported depression as an outcome measure (*n* = 10), the mean effect size was *g* = 0.20, 95% CI [−0.08, 0.48], which is considered a small effect (Lipsey & Wilson, 1993). The test for heterogeneity resulted in *Q* = 3.31, *p* = .914, *I* ^2^ = 0%.

### Publication Bias

Egger's test of the intercept resulted in *t*(13) = 0.84*, p* = .415, which shows no indication of publication bias. Duval and Tweedie's (2000) trim‐and‐fill method resulted in no missing studies. These analyses were repeated after removing one study (Descilo et al., 2009), which was an outlier in the meta‐analysis. Egger's test of the intercept resulted in *t*(12) = 1.31, *p* = .213, and the trim‐and‐fill method resulted in no missing studies, which shows no evidence of publication bias. The funnel plots including and excluding the study by Descilo et al. (2009) are presented in Supplementary Figures S1 and S2.

### Subgroup Analyses

For all subgroup analyses, the study by Descilo et al. (2009) was excluded as an outlier that would have largely influenced the analyses. The subgroup analysis of the studies with high (*n* = 9) versus low risk of bias (*n* = 5) resulted in a mean effect size *of g* = 0.70, 95% CI [0.26, 1.14] for the high‐risk studies and *g* = 0.39, 95% CI [0.16, 0.63] for low‐risk studies. There was no significant difference between the subgroups, *Q* = 1.41, *p* = .247.

The subgroup analysis comparing the studies with military participants (*n* = 5) versus studies with civilian participants (*n* = 8) resulted in a mean effect size of *g* = 0.36, 95% CI [0.11, 0.61] for the military studies and a mean effect size of *g* = 0.54, 95% CI [0.15, 0.92] for the civilian studies. No significant difference between the subgroups was found, *Q* = 1.57, p = .457. The study by Mitchell et al. (2014) was excluded from this analysis because it had both military and civilian participants.

The subgroup analysis comparing the studies with active (*n* = 7) versus waitlist control conditions (*n* = 7) resulted in a mean effect size of *g* = 0.30, 95% CI [0.09, 0.52] for the active condition studies and a mean effect size *g* = 0.61, 95% CI [0.18, 1.05] for the waitlist control studies. We found no significant difference between these subgroups, *Q* = 1.54, *p* = .215. The subgroup analysis comparing the studies with a yoga intervention (*n* = 6) with other body‐ and/or movement‐oriented interventions (*n* = 8) resulted in a mean effect size of *g* = 0.53, 95% CI [0.08, 0.99] for the yoga studies and a mean effect size of *g* = 0.35, 95% CI [0.15, 0.55] for the studies that included other BMOIs. There was no significant difference between the subgroups, *Q* = 0.52, *p* = .473.

The additional sensitivity analyses with *r* values of .5 and .3 resulted in mean effect sizes of *g* = 0.69, 95% CI [0.20, 1.18] for *r* = .5; and *g* = 0.60, 95% CI [0.15, 1.06] for *r* = .3. The sensitivity analysis calculating the effect size based on the posttreatment group difference resulted in a mean effect size of *g* = 0.68, 95% CI [0.18, 1.18]; the tests for heterogeneity resulted in values of *Q* = 124.4, *p* < .001 and *I* ^2^ = 90%. In this analysis, the study by Descilo et al. (2009) was again identified and removed as an outlier, which resulted in a mean effect size of *g* = 0.41, 95% CI [0.23, 0.59]. After this, heterogeneity tests resulted in values of *Q* = 13.8, *p* = .310, and *I* ^2^ = 13%. The additional analysis performed to calculate the mean effect size of RCTs only resulted in a mean effect size of *g* = 0.63, 95% CI [0.35, 0.92]. The test for heterogeneity resulted in *Q* = 27.3, p = .004, *I* ^2^ = 60%.

## Discussion

In this study, we examined the effectiveness of BMOIs in the treatment of PTSD in adults. In total, 22 studies were included in a systematic review, 15 of which met the inclusion criteria for the meta‐analysis. The meta‐analysis showed that BMOIs result in a significant decrease in PTSD symptoms, with medium‐to‐large mean effect sizes. The individual studies concerned different groups of patients with PTSD, such as veterans and individuals suffering from exposure to early childhood abuse, natural disasters, and traumatizing events in occupational settings. This heterogeneity in combination with the broad range of BMOIs may well be responsible for differences in treatment effects. From the additional subgroup analyses of the data, no significant differences between the subgroups emerged.

As individuals with PTSD often suffer from comorbid depression, and some reviews have indicated that BMOIs can alleviate depression severity (D'Silva, Poscablo, Habousha, Kogan, & Kligler, 2012; Luberto, White, Sears, & Cotton, 2013; Schuch et al., 2016), we also examined the effectiveness of BMOIs for comorbid depression. Only six studies included outcome measures for comorbid depression. The interventions contributed to a decrease in comorbid depressive symptoms, although the mean effect size was small.

One study was identified as outlier in the meta‐analysis (Descilo et al., 2009) and was therefore excluded from further analyses. This study showed a significantly larger effect size than the other studies in the analysis. An explanation for this larger effect size may be that the group of participants in this study, victims from natural disaster living in refugee camps, was distinctly different from the groups in the other studies, which consisted mainly of participants with PTSD resulting from early childhood trauma as well as veterans with combat‐related PTSD.

There is a large variety of BMOIs, and this kind of intervention is relatively novel in the treatment of PTSD. Therefore, cautiousness in the interpretation of this study's results is warranted given the preliminary nature of the review and the limited number of studies available on the topic of interest. However, from the studies included in this review, it can be concluded that both adding BMOIs to the usual (cognitive behavioral and/or pharmaceutical) treatments and providing BMOIs as stand‐alone treatment may result in a significant decrease of PTSD symptoms. The studies in this systematic review and meta‐analysis consisted of a heterogenic group of participants and a broad range of BMOIs. Therefore, conclusions concerning the effectiveness of specific BMOIs cannot be drawn from this study. The results of this study may give some support for the hypothesized mechanisms of regulating neurophysiological arousal (as described in the earlier) and may lay a foundation for more in‐depth studies on working mechanisms and effectiveness of specific interventions as well as on the effectiveness of interventions for specific subgroups of individuals with PTSD. Until now, some specific working mechanisms have been suggested. For instance, habituation to bodily sensations might play an important role (LeBouthillier, Fetzner, & Asmundson, 2016), possibly complemented by the experience of peaceful embodiment, a sense of ownership, and improvement of body awareness (LaChiusa, 2016; Rhodes, 2015). However, these mechanisms need to be studied more in depth, along with potential mediators and modulators.

Although further research on the effectiveness and working mechanisms of BMOIs is needed, the results of this study indicate that it may be useful to add BMOIs to established PTSD treatments. In fact, many people with PTSD currently make use of BMOIs despite a lack of evidence for their benefit (Meijnckens & Hesselink, 2016). As somatic and psychosomatic pain and other physical health problems often remain after established PTSD treatments (Galovski et al., 2009; Shipherd et al., 2014), patients may favor complementary therapies because they use an integrative approach to healing without manifesting side effects (Wahbeh, Senders, Neuendorf, & Cayton, 2014), thus alleviating not only PTSD symptoms but also enhancing physical health (Descilo et al., 2009; Gordon et al., 2016; Price, 2005) and alleviating symptoms of pain and fatigue (Kahn et al., 2016; Nakamura et al., 2017). From this perspective, adding BMOIs to established treatments could improve general treatment adherence and prevent premature dropout. This would be a valuable line of inquiry for future research.

The current systematic review and meta‐analysis were limited by the heterogeneity in terms of what could be classified as a BMOI and the relatively small number of studies available about the topic of interest. In view of the latter concern, a broad range of interventions, as well as smaller trials, pilot studies, and non‐randomized studies, were also included in order to reach a complete overview and enhance the total number of participants to draw conclusions from the meta‐analysis. From the 15 included studies, five studies were considered to have a low risk of bias, with no more than one unclear area in the Cochrane Risk of Bias tool. Although a moderate effect size was calculated from the results of these five studies, the number of high‐quality studies was small, and no definitive conclusions can be drawn. Other limitations of this study were the exclusion of gray literature, the consequences of this being that potentially interesting studies might have been missed along with the fact that this study was not preregistered.

In conclusion, BMOIs may be effective in reducing symptoms of PTSD and comorbid depression. More research is needed to specify which type of BMOIs are most effective for various subgroups of patients and which working mechanisms underlie these effects.

## Supporting information

Supporting Information
