# Supplementary material for: Body‐ and Movement‐Oriented Interventions for Posttraumatic Stress Disorder: A Systematic Review and Meta‐Analysis
Source: J Trauma Stress. 2019 Oct 28;32(6):967–76. doi: 10.1002/jts.22465 (PMC6973294; doi:10.1002/jts.22465)
Supplement: Supplementary file 1 — Supporting Information [file JTS-32-967-s001.docx]

Supplementary materials:

- Table S1: characteristics of selected studies
- Appendix 1: a full electronic search strategy
- Figure S1: funnel plot including the study by Descilo et al. (2009)
- Figure S2: funnel plot excluding the study by Descilo et al. (2009)

Table S1 Charateristics of selected studies

| **Author (year)** | **Sample size** | **Population (inclusion criteria)** | **Mean age at baseline** | **Study design** | **Description of conditions (interventions and controls)** | **Symptom severity: PTSD + depression measure**  **Mean and SD at baseline** | **Follow up period from post intervention** |
| --- | --- | --- | --- | --- | --- | --- | --- |
| McCarthy, Fuller, Davidson, Crump, Positano & Alderman (2017) | N = 28 | Australian veterans with combat related PTSD and intact cognitive functioning | M = 63.5 | Repeated measures with 8 week baseline period | Trauma sensitive yoga. 8 weekly sessions, aimed at body awareness, self-adaptation and empowerment. | PCL: M = 53.2, SD = 11.7  DASS depression: M = 17.4, SD = 8.5 | - (pre - post) |
| Nakamura, Lipschitz, Donaldson, Kida, Williams, Landward, et al. (2017) | N = 60 | Veterans who served in the Persian Gulf War | 1. M = 52.6  2. M = 49.2 | RCT with two arms | 1. Sleep Hygiene education: standard lectures and group discussions on sleep hygiene, focusing on tips to deal with difficulties sleeping. 3 weekly sessions  2 Mind body bridging: learning MBB concepts that assist in dealing with sleep problems. 3 weekly sessions. Sleep problems in PTSD are part of the PTSD symptomatology, according to DSM and therefore included. | 1. PCL-M: M = 49.6 [95% CI 43.6 - 55.7]  CES-D: M = 26.2 [95% CI 23.3 - 29.2]  2. PCL-M: M = 52.8 [95% CI: 47.5 - 58.0]  CES-D: M = 27.4 [95% CI 25.2 - 29.5] | 3 - months |
| Price, Spinazzola, Musicaro, Turner, Suvak, Emerson, et al. (2017) | N = 9 | Women with unresponsive chronic PTSD | M = 40.7 | Repeated measures | 20 weekly, one hour, trauma informed hatha yoga group sessions and 30 minute practice at home three times a week. | CAPS: M = 72.89, SD = 16.06 | 2 month |
| Walker & Pacik (2017) | N = 3 | Military veterans with PTSD | M = 68 | Case reports | Sudarshan Kriya (SKY) - Power breath workshop, a 5 day mind - body course, each day’s session is 3 - 4 hours. | PCL-M: M = 49.3 [Range 31 - 72] | - (pre - post) |
| Gordon, Staples, He & Atti (2016) | N = 92 | Palestinian adults with a DSM - IV PTSD diagnosis | M = 29.9 | Repeated measures design | Mind body skills group. Weekly, 2 hour group sessions. Meditation and training, practice and reflection on mind body skills. | HTQ: M = 2.8, SD = 0.3  Hopkins depression subscale: M = 2.7, SD = 0.5 | 11 months |
| Kahn, Collinge & Soltysik (2016) | N = 181 veterans  n = 139 non - veteran partners | Veteran - partner dyads deployed in post 9/11 combat operation | n.a. | 4 - arm RCT | 1.Mission Reconnect (MR): The 8 week program aims at — connecting with yourself, connecting with quiet, and connecting with your partner —together comprising 11 individual activities done at home. Instruction is provided by a 90 minute launch meeting, via videos, guided audio exercises, and written materials.  2.MR + Prevention and Relationship Enhancement Program (PREP): all dyads attended a weekend intervention aimed at communication and relationship building, problem solving, stress and relaxation, intimacy, forgiveness, and commitment. Afterwards they got the MR launch meeting.  3. PREP alone  4. waitlist control: 90 minute launch meeting with instruction to continue with usual behaviour regarding self - care or wellness related activity. | 1 PCL-C: M = 38.4, SD = 16.5  BDI: M = 14.5, SD = 10.8  2. PCL-C: M = 41.7, SD = 18.3  BDI: M = 16.1, SD = 12.5  3. PCL-C: M = 42.1, SD = 16.1  BDI: M = 19.5, SD = 12.0  4. PCL-C: M = 41.1, SD = 15.8  BDI: M = 16.8, SD = 12.7 | 2 months |
| Jindani, Turner & Khalsa (2015) | N = 60 | Canadian adults with PTSD (PCL score >57). | M = 41 | RCT with 2 arms | 1. Yoga: kundalini yoga, 8 weekly sessions of 90 minutes and 15 minute daily home practice. Yoga aimed at relaxation skills training, mindful awareness of the body mind and breath, balance, social integration.  2. Waitlist | 1. PCL 17: M = 59.9, SD = 9.3  DASS 21 (depression): M = 8.1, SD = 4.7  2. PCL17: M = 55.1, SD = 11.9  DASS 21 depression: M = 10.3, SD = 5.8 | - (pre - post) |
| Mitchell, Dick, DiMartine, Smith, Niles, Koenen & Street (2014) | N = 38 | Veteran and civilian adult women, age 18 - 65, positive on PC - PTSD | - | RCT with 2 arms | 1. Yoga group (n = 20), Hatha yoga, using guidelines for trauma - sensitive yoga, 12 weekly sessions or 12 session in 6 weeks, 75 minutes a session.  2. Waitlist control group, participants met once a week to complete questionnaires. Received 12 yoga sessions afterwards (n = 18) | 1.PCL: M = 51.94, SD = 14.36  CES-D: M = 29.58, SD = 16.01  2. PCL: M = 53.44, SD = 10.56  CES-D: M = 30.83, SD = 8.96 | 1 - month |
| Rosenbaum, Sherrington & Tiedemann (2014) | N = 81 | Men and women (Age >18yrs) with confirmed PTSD diagnosis, from PTSD in - patient unit. | 1. M = 47.1  2. M = 52.0 | RCT with 2 arms | 1. Usual care and 12 - week exercise intervention, weekly supervised exercise sessions, two similar unsupervised home - based exercise sessions and a walking program (with use of pedometer and exercise diary).  2. Usual care: combination of psychotherapy, pharmaceutical interventions, group therapy by psychologists. | 1. PCL-C: M = 64.5, SD = 11.9  DASS – depression: M = 24.8, SD = 11.5  2. PCL-C: M = 64.2, SD = 12.2  DASS – depression: M = 26.4, SD = 12.3 | - |
| Seppala, Nitschke, Tudorascu, Hayes, Goldstein, Nguyen, Perlman & Davidson (2014) | N = 21 | Male US veterans from Afghanistan or Iraq, age >18, English fluency | 1. M = 28.09 2. M = 29.20 | RCT with 2 arms | 1. Sudarshan Kriya yoga - breathing based meditation, 7 day intervention  2. Waitlist control group | 1. PCL-M: M = 36.55, SD = 11.44  MASQ-GDD: M = 23.00, SD = 8.04  2.PCL-M: M = 32.40, SD = 13.34  MASQ-GDD: M = 21.60, SD = 7.34 | 1 month and 1 year post intervention |
| Thordardottir, Gudmundsdottir, Zoega, Valdimarsdottir & Gudmundsdottir (2014) | N = 66 | Adults with self - reported distress or stress symptoms after earthquake, in Iceland, age 20 - 67. | - | Non - randomized controlled study | 1. Yoga intervention. Integrated hatha yoga program, 60 minute yoga sessions, twice a week. Physical activities of mild to moderate intensity in context of mindfulness and meditation, yogic breathing and relaxation.  2. waitlist control group | 1. PDS: M = 19.00, SD = 13.58  BDI-II: M = 15.60, SD = 9.19  2. PDS: M = 17.54, SD = 13.46  BDI-II: M = 17.20, SD = 11.42 | - (pre - post) |
| Van der Kolk, Stone, West, Rhodes, Emerson, Suvak & Spinnazola (2014) | N = 64 | Women, 18 - 58 years old, chronic treatment nonresponsive PTSD (having had >3 years of prior therapy for PTSD). Engaged in ongoing supportive therapy. | 1. M = 41.5  2. M = 44.3 | RCT with 2 arms | 1. 10 - week 1 hour protocoled trauma informed yoga class, incorporating basic elements of Hatha yoga: breathing, postures and meditation.  2. control condition: 10 week, 1 hour women’s health education (were offered free yoga classes for 10 weeks, free of charge after posttreatment evaluation) | 1. CAPS: M = 73.94, SD = 20.83  BDI-II: M = 20.89, SD = 11.13  2. CAPS: M = 76.66, SD = 20.83  BDI-II: M = 24.06, SD = 11.47 | - (pre - post) |
| Carter, Gerbarg, Brown, Ware, D’Ambrosio, Anand, Dirlea, Vermani & Katzman (2013). | N = 31 | Vietnam Veterans (male), all classified as disabled and using several psychofarmaca (i.e. SSRI, buspirone, atypical antipsychotics) | 1. M = 58.5  2. M = 58.4 | RCT with 2 arms | 1. Modification of Sudarshan Kriya Yoga (SKY) intervention, a yoga based stress reduction program, including psycho - education on stress - management, various breathing techniques, yoga poses, guided meditation and reflection. Administered over 5 consecutive days, 22hrs in total. Followed by weekly, 2hr follow - up group session for 1 month and one - monthly thereafter for a total of 6 months.  2. Waitlist, received intervention afterwards | 1. CAPS: M = 56.3, SD = 12.3  CES-D: M = 24.7, SD = 13.6  2. CAPS: M = 56.6, SD = 18.7  CES-D: M = 29.1, SD = 9.2 | 6 - weeks, 6 - months |
| Kim, Schneider, Bevans, Kravitz, Mermier, Qualls & Burge (2013) | N = 29 | Nurse volunteers (n = 28 women), >18yrs and employed at University of New Mexico Hospital. | 1. M = 47.6  2. M = 45.0  3. M = 44.6 | 3 - arm RCT, 3^rd^ arm included healthy (not diagnosed with PTSD) participants. | 1. Mind - body intervention: 16 standardized semi - weekly 60 - munite sessions. Consisting of stretching and balancing movements, breathing and a focus on control of attention and awareness.  2. Control group  3. BASE group: not diagnosed with PTSD | 1. PCL-C: M = 43.1, SD = 11.2  2. PCL-C: M = 42.6 SD = 12.7  3. PCL-C: M = 21.8 SD = 3.4 | - |
| Staples, Hamilton & Uddo (2013) | N = 12 | Veterans, previously diagnosed with PTSD (n = 10, male) | M = 62.2 | Single group, repeated measures | Yoga intervention, based on krishnamacharya yoga, linking breath to movement and using a specific meditative focus. 1 hour, twice a week for 6 weeks (12 sessions total) | PCL-M: M = 58.2 SD = 15.4 | -  End of intervention |
| Collinge, Kahn & Soltysik (2012) | N = 43 | Veterans and their partners | Veteran: M = 34  Partner: M = 29.3 | Single group, Repeated measures design | Mind - body interventions taught by audio CD, and massage for stress reduction taught by audio CD. Subjects were instructed to practice 3 - 4 times a week for period of 8 weeks. | PCL-C: veterans: M = 34.7 SD = 13.6  Partners: M = 31.8, SD = 11.1  BDI-II: veterans: M = 12.6, SD = 11.5  Partners: M = 10.1, SD = 7.8 | -  End of intervention |
| Nakamura, Lipschitz, Landward, Kuhn & West (2011) | N = 63 | Male and female US veterans with self reported sleeping problems assessed by MOS - SS | 1. M = 49.9  2. M = 53.8 | RCT with 2 arms | 1. Sleep hygiene program, two weekly sessions of 1h sleep education aimed at good sleeping practices.  2. Mind - body bridging program for sleep management, two weekly sessions, 1,5hrs. Aimed at identifying a possible cause of sleep difficulties from a mind - body perspective. Encouraged to practice daily around bedtime. | 1. PCL-M: M = 43.5, SD = 18.0  CES-D: M = 24.0 SD = 11.1  2. PCL-M: M = 42.2, SD = 16.6  CES-D: M = 24.5 SD = 12.2 | - (pre - post) |
| Hoekenga, Thewissen, Bos & Willemse - van Son (2010) | N = 31 | Adults with PTSD diagnosis based on DSM - IV - TR, expectance to need treatment for at least 3 months, capable to complete questionnaires in Dutch. | 1. M = 39.0  2. M = 39.4 | Quasi experimental, pre - post measures | 1. Treatment as usual + Body oriented therapy aimed at increasing feelings of control, adequate body - awareness, aggression regulation and feelings of safety in interaction with others, 12 weekly sessions n = 14  2. Treatment as usual (consisting of one or more of: EMDR, exposure, cognitive therapy, behavioural therapy, pharmacological treatment) n = 17 | 1.Dutch PTSD measure: ZIL  Hyperarousal: M = 3.34, SD = 0.50  Avoidance: M = 2.96, SD = 0.61  Intrusions: M = 3.08, SD = 0.85  2. ZIL  Hyperarousal: M = 3.24, SD = 0.59  Avoidance: M = 2.98, SD = 0.53  Intrusions: M = 3.29, SD = 0.62 | - |
| Kaiser, Gilette & Spinazzola (2010) | N = 10 | Adults with history of childhood interpersonal trauma | M = 46.7 | Randomized controlled two - group, repeated measures pilot study | 1. Sensory integration treatment - a sensory learning program, 30 day, multimodal program, using visual, acoustic and vestibular interventions.  2. Waitlist control group | 1. SIDES: M = 62.2, SD = 13.44  2. SIDES: M = 37.0, SD = 15.81 | 45 days after completion home light treatment* |
| Descilo, Vedamurtachar, Gerbarg, Nagajara, Gangadhar, Damodaran, Adelson, Braslow, Marcus & Brown (2009). | N = 183 | Native Tamil speakers, living in refugee camps. Women n = 160, men n = 23,  >18yrs old.  PCL score>50 | 1. M = 30.8  2. M = 35.1  3. M = 34.7 | Non - randomized repeated measures design | 1.Breath intervention  2.Breath intervention and exposure therapy (Traumatic incident reduction)  3. Wait list | PCL-17:  1. M = 66.5  2. M = 64.1  3. M = 67.9 | 6, 12, 24 weeks post intervention |
| Manger & Motta (2005) | N = 9 | Adults with PTSD, CAPS>20; not engaged in regular physical exercise 1 month before study; age 18 - 65; not actively suicidal | M = 48.1 | Single group, pre - post study | 10 week exercise program (10mn warming up; 30mn walking/jogging on treadmill with moderate intensity 60 - 80% max heartrate; 10mn cooling down), exercise twice a week. | Baseline 2:  CAPS: M = 56.67, SD = 22.51  PDS: M = 32.67, SD = 18.03  BDI-II: M = 16.33, SD = 11.90 | 1 - month |
| Price (2005) | N = 24 | Women with history of sexual abuse; age>25; engaged in psychotherapeutic relation for >2mnths; have a minimum of 2 years psychotherapy; not seeking bodywork during study involvement. |  | Two - group repeated measures, efficacy study | 1. Body oriented therapy as adjunct to psychotherapy, consisting of three phases. Phase 1 involved massage with verbalization of body sensations. In phase 2 addition of body awareness exercises. Phase 3: massage with body literacy and delving practice (similar to mindfulness meditation).  8, hour long sessions within a 10 week period.  2. standardized massage, adjusted protocol from Touch Therapy Institute in Miami. 8, hour long sessions within a 10 week period. | 1. Crime related PTSD scale:  M = 1.2, SD = 0.61  2. Crime related PTSD scale:  M = 1.0, SD = 0.61 | 1 month and 3 months |

Supplementary Materials: a full electronic search strategy.

1 sport therapy.mp.

2 physical education.mp.

3 physical training.mp.

4 physical recreation.mp.

5 physical therapy.mp.

6 physical activity.mp.

7 movement therapy.mp.

8 psychomotor therapy.mp.

9 running therapy.mp.

10 movement oriented psychotherapy.mp.

11 body oriented psychotherapy.mp.

12 dance movement therapy.mp.

13 dance therapy.mp.

14 psychomotricity.mp.

15 relaxation.mp.

16 yoga.mp.

17 Movement - oriented.mp.

18 Body - oriented therapy.mp.

19 Body awareness therapy.mp.

20 Body attitude.mp.

21 Body experience.mp.

22 Body cathexis.mp.

23 Body scheme.mp.

24 Body image.mp.

25 Breath therapy.mp.

26 Body relatedness.mp.

27 Psychomotor.mp.

28 Bodypsychotherapy.mp.

29 Complementary therapies.mp.

30 Experiential therapies.mp.

31 Physical awareness.mp.

32 Body consciousness.mp.

33 Integrative Movement therapy.mp.

34 Tai chi.mp.

35 Chi Kung.mp.

36 chi gung.mp.

37 Functional Relaxation.mp.

38 Body therapy.mp.

39 Embodiment.mp.

40 Sensory integration.mp.

41 Sensorimotor.mp.

42 Exercise.mp.

43 Arousal regulation.mp.

44 Stress inoculation training.mp.

45 Stress management.mp.

46 Pesso.mp.

47 Body - mind.mp.

48 Mind - body.mp.

49 1 or 2 or 3 or 4 or 5 or 6 or 7 or 8 or 9 or 10 or 11 or 12 or 13 or 14 or 15 or 16 or 17 or 18 or 19 or 20 or 21 or 22 or 23 or 24 or 25 or 26 or 27 or 28 or 29 or 30 or 31 or 32 or 33 or 34 or 35 or 36 or 37 or 38 or 39 or 40 or 41 or 42 or 43 or 44 or 45 or 46 or 47 or 48

50 Psychological Trauma.mp.

51 Psychotrauma.mp.

52 Traumatic Stress.mp.

53 PTSD.mp.

54 Posttraumatic Stress.mp.

55 Violence.mp.

56 Abuse.mp.

57 Torture.mp.

58 Veterans.mp.

59 Sexual Trauma.mp.

60 Sexual Violence.mp.

61 Sexual Abuse.mp.

62 50 or 51 or 52 or 53 or 54 or 55 or 56 or 57 or 58 or 59 or 60 or 61

63 49 and 62

*Figure S1*. Funnel plot including the study by Descilo et al. (2009)

*Figure S2*. Funnel plot excluding the study by Descilo et al. (2009)
